# Brain activity in response to food images in patients with irritable bowel syndrome and functional dyspepsia

**DOI:** 10.1007/s00535-023-02031-5

**Published:** 2023-08-12

**Authors:** Ryo Katsumata, Takayuki Hosokawa, Noriaki Manabe, Hitoshi Mori, Kenta Wani, Katsunori Ishii, Tomohiro Tanikawa, Noriyo Urata, Maki Ayaki, Ken Nishino, Takahisa Murao, Mitsuhiko Suehiro, Minoru Fujita, Miwa Kawanaka, Ken Haruma, Hirofumi Kawamoto, Toshihiro Takao, Tomoari Kamada

**Affiliations:** 1 Department of Health Care Medicine, Kawasaki Medical School General Medical Center, 2-6-1, Nakasange, Kita-Ku, Okayama, 700-8505 Japan; 2 Department of Orthoptics, Faculty of Rehabilitation, Kawasaki University of Medical Welfare, 288 Matsushima, Kurashiki, Okayama, 701-0193 Japan; 3 Department of Clinical Pathology and Laboratory Medicine, Kawasaki Medical School General Medical Center, 2-6-1, Nakasange, Kita-Ku, Okayama, 700-8505 Japan; 4 Department of Neurology, Kawasaki Medical School General Medical Center, 2-6-1, Nakasange, Kita-Ku, Okayama, 700-8505 Japan; 5 Department of Psychiatry, Kawasaki Medical School General Medical Center, 2-6-1, Nakasange, Kita-Ku, Okayama, 700-8505 Japan; 6 Department of General Internal Medicine 2, Kawasaki Medical School General Medical Center, 2-6-1, Nakasange, Kita-Ku, Okayama, 700-8505 Japan; 7 Department of Health Care Medicine, Kawasaki Medical School, 577, Matsushima, Kurashiki, 701-0192 Japan

**Keywords:** Functional dyspepsia, Gut–brain axis, Irritable bowel syndrome, Near-infrared spectroscopy, Prefrontal cortex

## Abstract

**Background:**

Functional dyspepsia (FD) and irritable bowel syndrome (IBS) are caused and exacerbated by consumption of fatty foods. However, no study has evaluated brain activity in response to food images in patients with disorders of gut–brain interaction (DGBI). This study aimed to compare food preference and brain activity when viewing food images between patients with DGBI and healthy controls.

**Methods:**

FD and IBS were diagnosed using the ROME IV criteria. Food preference was assessed using a visual analog scale (VAS). Brain activity in the prefrontal cortex (PFC) in response to food images was investigated using functional near-infrared spectroscopy (fNIRS).

**Results:**

Forty-one patients were enrolled, including 25 with DGBI. The mean VAS scores for all foods (controls vs. FD vs. IBS: 69.1 ± 3.3 vs. 54.8 ± 3.8 vs. 62.8 ± 3.7, *p* = 0.02), including fatty foods (78.1 ± 5.4 vs. 43.4 ± 6.3 vs. 64.7 ± 6.1, *p* < 0.01), were the lowest in patients with FD among all groups. Patients with FD had significantly higher brain activity in the left PFC than those with IBS and healthy controls (mean *z*-scores in controls vs. FD vs. IBS: − 0.077 ± 0.03 vs. 0.125 ± 0.04 vs. − 0.002 ± 0.03, *p* < 0.001).

**Conclusions:**

Patients with DGBI, particularly those with FD, disliked fatty foods. The brain activity in patients with DGBI differed from that in healthy controls. Increased activity in the PFC of patients with FD was confirmed.

**Supplementary Information:**

The online version contains supplementary material available at 10.1007/s00535-023-02031-5.

## Introduction

Functional dyspepsia (FD) and irritable bowel syndrome (IBS) are major disorders of gut–brain interaction (DGBI), with huge social impact because of their long-lasting symptoms that lead to decreased quality of life (QOL) and labor productivity [1, 2]. The prevalence of FD and IBS in Japan was reportedly 2.4% and 2.2%, respectively [3]; these conditions can be considered common diseases that require intervention. FD and IBS share similar pathophysiology, including impaired motility, influence of food, micro-inflammation, elevated mucosal permeability, psychosocial factors, and disrupted gut–brain interaction [4]. Additionally, psychological comorbidities, such as depression and anxiety disorders, are more frequently detected in patients with FD and those with IBS than in the general population [5, 6].

Fatty food intake can trigger gastrointestinal (GI) symptoms in patients with FD [7] and IBS [8]. However, other food types, such as carbohydrates, do not induce such symptoms and are considered acceptable for patients with FD [7], which indicates that the type of food is a crucial factor for GI symptoms in those with DGBI. To date, differences in preference for various food images in patients with FD and those with IBS have been scarcely elucidated, and to the best of our knowledge, no study has reported on disparities between preferences for fatty and light foods.

Brain activity in patients with DGBIs has received increasing attention recently. Previous studies have reported activity in several brain regions associated with pain processing in patients with FD compared with that in healthy controls at resting state [9], as well as activity in pain-associated brain regions in response to rectal distention in those with IBS [10]. Notably, the prefrontal cortex (PFC) is involved in emotion suppression and decision-making, and impairment of PFC function has been reported in various mental disorders [11]. Furthermore, alteration in brain activity, including activity in the PFC, when receiving intestinal dilatation in patients with FD and those with IBS, who have similar brain response to that in healthy controls, has been confirmed [12–14]. However, no study has been published on activity in the PFC in patients with FD and those with IBS in response to food images. Furthermore, a direct comparison of brain activity between FD and IBS has scarcely been reported.

Functional near-infrared spectroscopy (fNIRS) is a non-invasive modality for assessing real-time brain activity by estimating the alterations in oxygenated hemoglobin (oxy-Hb) concentrations in the cerebral cortex [15]. The results obtained from fNIRS have been proven to positively correlate with those of functional magnetic resonance imaging (fMRI) [16, 17]. Previous studies have reported the use of fNIRS, to evaluate brain activity during cognitive tasks and by emotion recognition in the PFC region [18, 19]. fNIRS has been used to evaluate hemodynamic response to cognitive tasks in patients with mental disorders, including schizophrenia and major depression [11, 20]. Although brain activity detected using fNIRS in GI disorders has been reported [21], a study assessing brain activity using fNIRS in patients with FD and those with IBS, who are considered to have altered brain activity compared with brain activity in healthy controls, is yet to be reported.

Therefore, this clinical study aimed to assess and compare the preference for various foods in patients with FD, those with IBS, and healthy controls and to evaluate brain activity in response to food images in the PFC using fNIRS. We hypothesized that the preference for food images and altered brain activity is lower in patients with DGBIs than in controls.

## Methods

This prospective cross-sectional study was conducted at the Kawasaki Medical School General Medical Center and Kawasaki University of Medical Welfare and was approved by the Research Ethics Committee of Kawasaki Medical School Hospital (IRB No. 5696-01). The study was also conducted in accordance with the Declaration of Helsinki. Written informed consent was obtained from all participants. We adhered to the guidelines of the Society for Functional Near-Infrared Spectroscopy for the fNIRS study [22].

The primary endpoint was differences in brain activity between patients with FD, those with IBS, and healthy controls. The secondary endpoint was differences in food preference among the three groups.

In this study, we did not perform power analysis before enrollment, as no clinical data were available for the fNIRS study in patients with DGBIs. Alternatively, post hoc power analysis was conducted to confirm the study power and estimated sample size with statistical power as at least 0.8 and *α* as 0.05. If the statistical power was < 0.8, we planned to enroll participants until the power exceeded 0.8. This study’s smallest sample size was initially set as 12 participants in each of the 3 groups, according to previous studies that evaluated emotion processing using fNIRS [23, 24].

### Participants

We included outpatients with FD and those with IBS diagnosed and subtyped using ROME IV criteria [25, 26] at Kawasaki Medical School General Medical Center from August 2022 to March 2023. Patients with FD or IBS were defined as those with DGBIs in this study, whereas healthy controls were participants without GI symptoms or current history of GI disorders. The exclusion criteria were as follows: age < 20 and > 65 years and participants with malignant disease, active *Helicobacter pylori* infection, iron deficiency anemia, diverticulosis, and a history of abdominal surgery or childhood abuse. Blood tests were performed in all patients to exclude anemia and elevated serum amylase levels. The active *H. pylori* infection status was assessed using serum antibody test, urea breath tests, or endoscopic findings. Regarding diagnosis of *H. pylori* infection using endoscopic findings, at least two gastroenterologists qualified as Board Certified Gastroenterologist of The Japanese Society of Gastroenterology and Board Certified Fellow of the Japan Gastroenterological Endoscopy Society agreed with the diagnosis. Upper GI endoscopy was performed in all patients with FD, and colonoscopy or abdominal computed tomography was performed in those with IBS to evaluate organic disorders, including symptomatic uncomplicated diverticular disease. Participants with a history of allergic reactions, such as skin rash, after consuming a particular food were defined as those with food hypersensitivity positivity.

### Images

We originally selected a wide variety of 40 food images, including those of fatty and light foods, as well as 20 animal images for control stimuli (Supplementary Fig. 1). Overall, 40 healthy participants with no GI symptoms or active GI disorders were asked to rate the grades of food fattiness using the visual analog scale (VAS). Foods ranked in the top 10 and those containing at least 20 g of fat per 100 g were defined as fatty foods, whereas those ranking in the last 10 and those containing at most 4 g of fat per 100 g, were defined as light foods.

### Symptoms and food preference

**Fig. 1:**
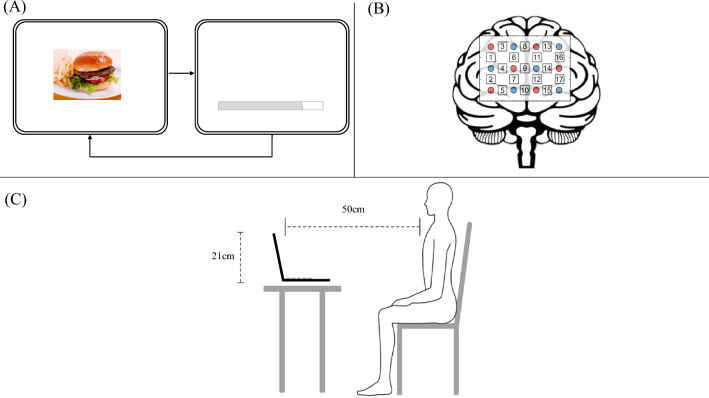
Procedure and experimental setting for this study. (**A**) Procedure for displaying images of food and animals used to evaluate food preference and brain activity. (**B**) Schematic orientation of the fNIRS probes superimposed on the graphic brain surface. Red circles represent the emitter probes, whereas blue circles represent the detector probes. The numbers indicate the channel number. (**C**) Experimental setup. The participants are in a bright quiet room, as shown in the figure. *fNIRS* functional near-infrared spectroscopy

GI symptoms were evaluated in all participants using the GI Symptom Rating Scale (GSRS), which contained 15 questions regarding digestive complaints. All questions and five major symptoms (reflux, abdominal pain, indigestion, diarrhea, and constipation) were scored on a scale of 1 to 7 [27]. Food preference and animal images displayed at the center of the laptop monitor were assessed using the VAS, with ratings ranging from not preferable (1) to preferable (100) (Fig. 1A).

### Measurements and analysis of fNIRS

Oxygenation changes in PFC were evaluated using Spectratech OEG-17APD (Spectratech Inc., Yokohama, Japan) to access relative oxy-Hb, deoxyhemoglobin (deoxy-Hb), and total hemoglobin (total-Hb) concentrations using two-wavelength lights (770 nm and 840 nm) based on the modified Beer–Lambert law [28]. According to the 10–10 electrode placement system, we attached and fixed the fNIRS probes to the forefront using an elastic band, with the lowest probe line located on Fpz (Fig. 1B). In this study, a total of 17 channels were measured using a 3 × 4 probe arrangement with a 3-cm inter-optode distance. These 17 channels are set on the PFC region, with areas at channels 1, 2, 3, 4, 6, 7, 11, 12, 13, 14, 16, and 17 approximately corresponding to the dorsolateral PFC (DLPFC), those at channels 8 and 9 corresponding to the medial PFC, and those at channels 5, 10, and 15 corresponding to the orbitofrontal cortex [29–31] (Supplementary Fig. 2).

### Visual stimuli experiment

We performed a block design using visual stimuli and resting state as the task and control, respectively. All task procedures were explained to all participants before the experiment. Participants sitting on a chair relaxed in a well-lit quiet room were placed in front of a laptop monitor (Fig. 1C). The distance between the participants and the monitor was 50 cm, and the size of the monitor was 21 cm, indicating a visual angle of 22.8°.

After applying the probes, a small plus sign was displayed for 30 s as the baseline period. Subsequently, each food image was randomly displayed, followed by a bar showing the remaining time to the next image (Fig. 1A). All procedures were performed using the PsychoPy software version 2022.1.3 (open-source software developed by the support of The Royal Society, The University of Nottingham, Wellcome Trust, and BBSRC, London, Nottingham, London, Swindon, United Kingdom) [32]. Overall, 40 food images were shown to the participants in this study. The image presentation duration and inter-trial interval were 7 and 10 s, respectively. Therefore, the participants were instructed to avoid gross movement during the experiment to minimize motion artifacts. Experimenters were retained out of the participants’ sight during the process.

### Data analysis of fNIRS

We set the sampling rate of the fNIRS measurement as 12.2 Hz, and the raw data were processed with a 0.01–0.3-Hz band pass filter. A hemodynamic signal separation method, according to a previous study [33], was used to reduce the effects of systemic physiological signals, including those of the skin surface. Subsequently, linear detrending was applied to all fNIRS signals. We used the oxy-Hb components during each presentation period in the analysis. *Z*-scores of each image were used to compare the brain activity, which was calculated by subtracting the mean oxy-Hb values from an individual value and subsequently dividing by the standard deviation of the values in the resting state. *Z*-scores  ˂ − 4 and ˃ 4 were considered outliers and were excluded from the analysis [34]. Motion artifacts, which are defined as abnormal values with or after the participants’ movements, were removed after the consensus of two experimenters (R. K and T. H).

### Statistical analysis

Normally distributed continuous variables are expressed as mean and standard deviation, whereas categorical data are presented as median and interquartile range. Frequencies are expressed as the number of cases and percentage. Student’s t test was used to compare continuous variables between the two groups. The *χ*^2^ test was used to compare the frequencies between the two groups and among the three groups. The Mann–Whitney *U* test was used to compare the categorical data between the two groups. Furthermore, comparisons among the three groups regarding the continuous data were conducted using analysis of variance (ANOVA) and two-way ANOVA with the Tukey–Kramer test as post hoc analysis. In the two-way ANOVA, one factor was food type (fatty or light food), and the other was clinical background (two levels: healthy controls or patients with DGBI, otherwise three levels: healthy controls, patients with FD, and those with IBS). Comparisons among the three groups regarding categorical data were performed using the Kruskal–Wallis test. All statistical analyses were performed using MATLAB (MathWorks Inc., Natick, MA, United States), and two-sided *p*-values < 0.05 were considered statistically significant.

## Results

### Clinical background

Overall, 41 participants, including 12 patients with FD and 13 with IBS, were enrolled in the study. Weight loss of 5% in the last 6 months, nocturnal abdominal pain, fever, and rectal bleeding were not observed in any of the patients. Among the 12 patients with FD, 4, 6, and 2 with epigastric pain syndrome (EPS), postprandial distress syndrome (PDS), and EPS and PDS, respectively, were included. Patients with both IBS and FD were enrolled in the IBS group in this study because they predominantly showed IBS-related symptoms. Among the 13 patients with IBS, 4 had constipation-predominant IBS (IBS-C), 6 diarrhea-predominant IBS (IBS-D), and 3 mixed IBS (IBS-M). The mean disease durations of FD and IBS were 6.83 and 10.07 years, respectively. Supplementary Table 1 presents the treatments and comorbidities in each group.

Demographic data, including age, sex proportion, body mass index, and frequencies of food allergy, alcohol consumption, and smoking status, were not significantly different between healthy controls and patients with DGBI (Table 1), as well as among healthy controls, patients with FD, and those with IBS (Table 2). Supplementary Table 1 shows the profiles regarding the subtypes of patients with FD and those with IBS, comorbidities, and current treatment.

**Table 1:** Comparison of clinical background and gastrointestinal symptoms between healthy controls and patients with disorder of gut–brain interaction

Variables	Control (*n* = 16)	DGBI (*n* = 25)	*p-*values
Age (mean [SD], year)	43.6 (3.3)	38.7 (2.6)	0.367^a^
Male Sex (*n* [%])	5 (31.3)	8 (32.0)	0.959^b^
BMI (mean [SD], kg/m^2^)	22.8 (0.8)	22.3 (0.7)	0.667^a^
Alcohol: current drinker (*n* [%])	6 (37.5)	7 (28.0)	0.825^b^
Smoking (*n* [%])	0 (0)	0 (0)	
Food allergy (*n* [%])	3 (18.7)	7 (28.0)	0.508^b^
Disease duration (mean [SD], year)	0 (0)	7.88 (6.3)	
GSRS score (median [IQR])			
Reflux	1.0 (1.0–1.0)	2.0 (1.5–2.5)	< 0.001^c^
Abdominal pain	1.0 (1.0–1.0)	2.3 (2.0–3.0)	< 0.001^c^
Indigestion	1.0 (1.0–1.2)	3.0 (2.2–4.1)	< 0.001^c^
Diarrhea	1.0 (1.0–1.0)	3.0 (1.7–3.7)	< 0.001^c^
Constipation	1.2 (1.0–1.7)	2.3 (2.0–3.3)	< 0.001^c^

*DGBI* disorder of gut–brain interaction, *SD* standard deviation, *n* number, *BMI* body mass index, *GSRS* gastrointestinal symptom rating scale, *IQR* interquartile range

^a^*p*-values were calculated using Student’s *t*-test, ^b^chi-square test, or ^c^Kruskal–Wallis test

**Table 2:** Comparison of clinical background and gastrointestinal symptoms among healthy controls, patients with functional dyspepsia, and those with irritable bowel syndrome

Variables	Control (*n* = 16)	FD (*n* = 12)	IBS (*n* = 13)	*p-*values
Age (mean [SD], year)	43.6 (3.3)	36.5 (3.8)	40.7 (3.6)	0.418^a^
Male sex (*n* [%])	5 (31.3)	4 (33.3)	4 (30.8)	0.965^b^
BMI (mean [SD], kg/m^2^)	22.8 (0.8)	21.2 (1.0)	23.2 (0.9)	0.362^a^
Alcohol: current drinker (*n* [%])	5 (31.3)	3 (25.0)	4 (30.8)	0.904^b^
Smoking (*n* [%])	0 (0)	0 (0)	0 (0)	
Food allergy (*n* [%])	3 (18.7)	3 (25.0)	4 (30.8)	0.624^b^
Disease duration (mean [SD], year)	0 (0)	6.83 (4.6)	10.07 (7.0)	0.213^c^
GSRS score (median [IQR])				
Reflux	1.0 (1.0–1.0)	2.3^*^ (2.0–3.5)	1.0 (1.0–1.2)	< 0.001^c^
Abdominal pain	1.0 (1.0–1.0)	2.3^**^ (2.0–3.7)	2.3^**^ (2.0–2.8)	< 0.001^c^
Indigestion	1.0 (1.0–1.2)	3.0^**^ (2.5–3.7)	3.0^**^ (2.1–4.1)	< 0.001^c^
Diarrhea	1.0 (1.0–1.0)	2.3^**^ (1.3–2.8)	3.3^**^ (2.1–5.0)	< 0.001^c^
Constipation	1.2 (1.0–1.7)	2.5^**^ (1.7–2.9)	3.0^**^ (2.0–4.0)	< 0.001^c^

*FD* functional dyspepsia, *IBS* irritable bowel syndrome, *SD* standard deviation, *n* number, *BMI* body mass index, *GSRS* gastrointestinal symptom rating scale, *IQR* interquartile range

^a^*p*-values were calculated using analysis of variance (ANOVA), ^b^chi-square test, ^c^Student’s *t* test between FD and IBS or Kruskal–Wallis test with Bonferroni correction method as a pairwise comparison

^*^p < 0.05/3, comparison with healthy control and patients with IBS, ^**^p < 0.05/3, comparison with healthy control

The mean GSRS scores for all five major symptoms were higher in the DGBI group than in the healthy control group (Table 1). The mean scores for reflux, abdominal pain, indigestion, and constipation were higher in patients with FD than in healthy controls, whereas the scores for abdominal pain, indigestion, diarrhea, and constipation were higher in patients with IBS than in healthy controls (Table 2).

### Food preference and animal images

**Fig. 2:**
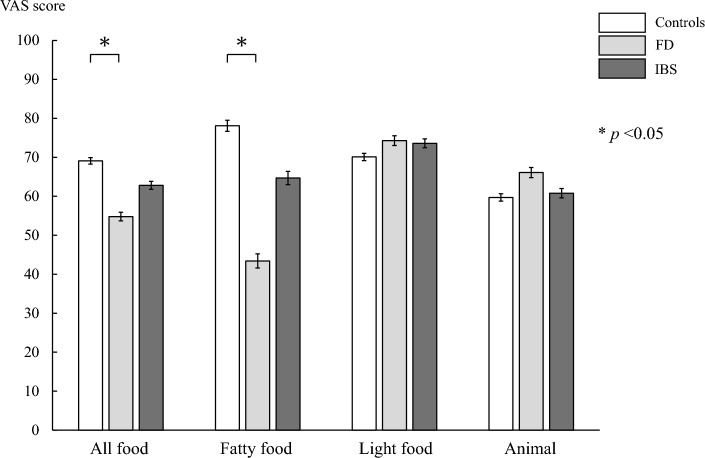
Comparison among preferences for images are shown in mean VAS scores using ANOVA with the Tukey–Kramer test as post-hoc analysis. Bar plots and error bars represent mean VAS scores and standard errors in each group. *FD* functional dyspepsia, *IBS* irritable bowel syndrome, *VAS* visual analog scale, *ANOVA* analysis of variance

The mean VAS scores for all foods (healthy controls vs. patients with DGBI: 69.1 ± 3.3 vs. 58.9 ± 2.7, *p* = 0.02), and those for fatty foods (78.1 ± 5.7 vs. 54.5 ± 4.6, *p* < 0.01), were lower in patients with DGBI than in healthy controls. However, scores for light food and animals did not differ significantly between the groups (Supplementary Fig. 3). As shown in Fig. 2, the mean VAS scores for all foods (healthy controls vs. patients with FD vs. those with IBS: 69.1 ± 3.3 vs. 54.8 ± 3.8 vs. 62.8 ± 3.7, *p* = 0.02), including fatty foods (78.1 ± 5.4 vs. 43.4 ± 6.3 vs. 64.7 ± 6.1, *p* < 0.01), were the lowest in patients with FD among the three groups. Multiple comparisons showed that the mean VAS score was significantly lower in patients with FD than in healthy controls. Furthermore, no significant differences were found in light food or animals among the three groups.

### Brain activity

**Fig. 3:**
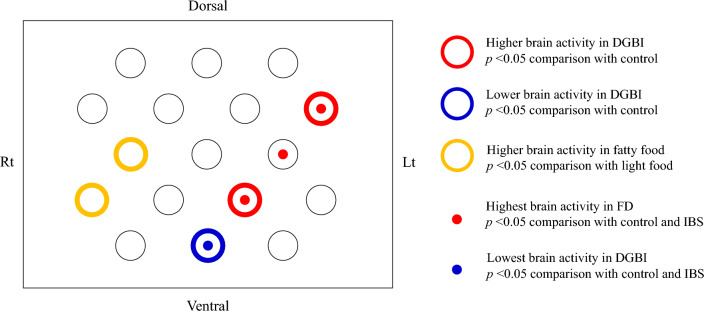
Areas corresponding to significant differences between patients with DGBI and healthy controls or among patients with FD, those with IBS, and healthy controls. Thick outer edges indicate significant differences between the two groups using Student’s *t*-test, and central circles indicate significant differences among the three groups using ANOVA with the Tukey–Kramer test as a post-hoc analysis. The red outer edges correspond to a higher brain activity in patients with DGBI than in healthy controls, and blue edge represents lower brain activity in patients with DGBI than in healthy controls. Yellow outer edges correspond to a higher brain activity in response to the fatty food images than to the light food images. Red dots in the centers show the highest brain activity in patients with FD among the three groups, and the blue dot in the center shows the lowest brain activity in patients with FD among the three groups. *DGBI* disorder of gut–brain interaction, *FD* functional dyspepsia, *IBS* irritable bowel syndrome

Initially, we removed 1.3% of data due to motion artifacts and then removed 0.2% of data due to outliers (see Data analysis of fNIRS in Methods). Figure 3 shows the locations of the channels with statistically significant results from the comparisons of z-scores. ANOVA showed the main effects of food type (channels 2 and 4) and clinical background (channels 10, 12, 14, and 16), whereas no significant interaction effect was found in the analysis between healthy controls and patients with DGBI, as well as among the three groups.

**Fig. 4:**
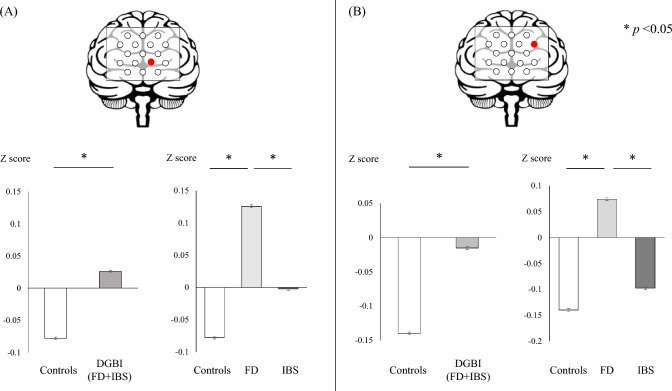
Comparison of brain activity. Data in channel 12 (**A**) and channel 16 (**B**) are presented. Data are shown for comparison of *z*-scores using Student’s *t*-test in the left dorsolateral PFC between controls and patients with DGBI (graphs on the left side for each channel) and comparison of *z*-scores using ANOVA with the Tukey–Kramer test as post hoc analysis in the left dorsolateral PFC among healthy controls, patients with FD, and those with IBS (graphs on the left side for each channel). *DGBI* disorder of gut–brain interaction, *FD* functional dyspepsia, *IBS* irritable bowel syndrome, *PFC* prefrontal cortex, *ANOVA* analysis of variance

Brain activity in the right DLPFC was greater when watching fatty foods than when watching light foods (at channel 2, light food vs. fatty food: − 0.11 ± 0.03 vs. − 0.01 ± 0.03, *p* = 0.04). On the other hand, the mean z-score in the left PFC (channel 12) was significantly higher in patients with DGBI than in healthy controls (healthy controls vs. patients with DGBI: − 0.07 ± 0.03 vs. 0.05 ± 0.02, *p* = 0.001) (Fig. 4A left graph). In the same area, among healthy controls, patients with FD, and those with IBS, the mean *z*-scores in patients with FD were the highest (controls vs. FD vs. IBS: − 0.077 ± 0.03 vs. 0.125 ± 0.04 vs. − 0.002 ± 0.03, *p* < 0.001) (Fig. 4A right graph). Similarly, in channel 16, the mean *z*-score was significantly higher in patients with DGBI than in controls (healthy controls vs. patients with DGBI: − 0.14 ± 0.04 vs. − 0.01 ± 0.03, *p* = 0.01) (Fig. 4B left graph), and the score was the highest in patients with FD among the three groups (healthy controls vs. patients with FD vs. those with IBS: − 0.14 ± 0.04 vs. 0.073 ± 0.04 vs. − 0.09 ± 0.04, respectively, *p* < 0.001) (Fig. 4B right graph). Furthermore, in channel 10, the mean *z*-score was lower in patients with DGBI than in healthy controls (healthy controls vs. patients with DGBI: 0.12 ± 0.05 vs. − 0.05 ± 0.04, *p* = 0.005), and the score was the lowest in patients with FD among the three groups (healthy controls vs. patients with FD vs. those with IBS: 0.12 ± 0.05 vs. − 0.09 ± 0.06 vs. − 0.01 ± 0.05, *p* = 0.012).

In this study, the statistical power was 0.837, as determined by post-hoc power analysis. The estimated smallest sample size for this study with statistical power of 0.808 and α of 0.05 was 10 participants in each group.

## Discussion

In this study, we initially presented brain activity in response to food images detected using fNIRS in patients with FD and those with IBS, as well as the different preferences for food images between patients with FD and those with IBS. Moreover, we detected the differences in brain activity in the right DLPFC when watching fatty and light food images and confirmed significantly higher left prefrontal activity in patients with FD than in healthy controls and those with IBS.

Notably, the preference for food images differed between patients with FD and those with IBS, which represented a lower score of fatty food images in the FD group than in the IBS and healthy control groups. Previous studies have shown that patients with FD have a lower rating score for high-fat food images than do healthy controls [35]. However, compared with healthy controls, patients with IBS were reported to show a higher consumption of meat [36] and have normal weight or be overweight [8]. Notably, these data are consistent with our results; however, the reasons for patients with IBS showing relatively higher emotional preferences toward fatty food than those with FD remain unclear. Therefore, we hypothesized that an immediate symptom onset to fatty food intake is associated with negative emotions in patients with FD. However, patients with IBS experienced GI symptoms such as diarrhea slightly after consuming fatty food, resulting in weaker negative emotion to fatty food than that in patients with FD. Indeed, the inflow of fat into the duodenum promptly induces GI symptoms such as nausea [37]. Psychologically, conditioned taste aversion (CTA) is a type of classical conditioning that strongly induces negative emotions, and the incidence of CTA is higher when the interval between food consumption and onset of illness is shorter than when the interval is longer [38]. Simultaneously, fear memories are attenuated by distraction during the consolidation process [39]. Therefore, our results may be explained by the difference in timing of symptom onset between FD and IBS and the mechanism of taste aversive learning. In this study, most participants with FD reported that when they watched fatty food images, they remembered abdominal symptoms such as abdominal pain and fullness from their experience, resulting in perceived discomfort. In contrast, only few participants with IBS reported perceived discomfort after watching fatty food.

Notably, increased activity in the PFC in patients with FD in response to food images was confirmed, regardless of the type of food. In a previous study using H_2_^15^O- positron emission tomography (PET), PFC was more activated by gastric balloon distention in non-abuse patients with FD than in abuse patients with FD [13]. Vandenberghe et al. reported that PFC activation during gastric balloon distention occurred at a lower threshold in patients with FD than in healthy controls [12]. This indicates that PFC hyperactivation is related to aversive stimulation in patients with FD. Since we enrolled non-abuse patients with FD, our results, which show hyperactivation of the PFC in food images, are consistent with previously published data detected using PET [13]. Additionally, depressive and anxious mental states in patients with FD may be related to this change. Patients with FD show a higher prevalence of depression and anxiety disorders than healthy individuals do [5], and a certain number of these patients, including those in our study, had a history of using antidepressants. Previously, hyperactivation of the DLPFC was observed in patients with anxiety disorders [40]. DLPFC, where brain activation was observed in our patients with FD, is believed to be associated with cognitive demands, including future prediction and expectation [41], attention to various information [42], and taste-associated activity [43]. Although the exact reflection of brain activation in DLPFC is not completely understood, common features between FD and mental disorders regarding brain abnormalities can provide insights into understanding patients’ experience and pathophysiology of FD. Furthermore, based on previous data showing that DLPFC deactivation was associated with pain relief [44], the alteration we confirmed can be a crucial marker for patients’ symptoms and effective treatment targets.

Regarding brain activity analysis, we did not detect clear characteristics between patients with IBS and healthy controls. In a meta-analysis, patients with IBS were confirmed to have decreased PFC activity during rectal distention [10]. Another study reported decreased activity in the PFC of patients with IBS during cognitive tasks [45]. However, in their study, patients with IBS showed different behavioral data, such as higher Nelson perseverative errors. Likewise, significantly higher subjective pain scores and altered brain activity in patients with IBS than in healthy controls with the same rectal distention volume were reported in another study [46]. On the contrary, our study’s task, which involved showing patients images of food, resulted in behavioral data that did not significantly differ between patients with IBS and healthy controls. This implies similar brain activity between patients with IBS and healthy controls. Therefore, other tasks with different behavioral responses are required to provide further insight into understanding brain activity in patients with IBS.

A previous study has shown that the primate orbitofrontal cortex is activated by fat intake compared with that by other taste stimuli, such as glucose [47]. In a human study, the right DLPFC was activated during a tasting task using glucose and salt [43]. Furthermore, sourness has been found to activate the right DLPFC activity [48]. Therefore, our results are consistent with past data and provide additional evidence that fattiness is a crucial factor in enhancing human prefrontal activity.

This study has some limitations. First, it was conducted at a single center with participants having similar genetic and ethnic backgrounds. Nevertheless, our data provide insights into the pathophysiology of FD and IBS in Japanese patients, which is crucial in obtaining specific data for each country and enhancing our understanding of the disease mechanism. Therefore, to provide a comprehensive understanding of the pathophysiology of DGBI, including FD and IBS, multi-center, international, large-scale studies with multi-ethnic participants should be conducted. Second, the group of patients with FD and those with IBS was heterogeneous regarding clinical backgrounds, such as subtypes, disease duration, and treatment. Therefore, since recruiting participants with a homogenous background was quite challenging and time-consuming, our initial approach in this study was to focus on detecting the characteristics of patients with FD and those with IBS as a whole. Despite the heterogeneous features in each group, significant differences were found among the three groups in terms of food preferences and brain activity in our study. This means that the characteristics are common and fundamental in patients with FD and those with IBS, regardless of the background, including disease duration, treatment, and subtypes. Therefore, to elucidate the detailed pathophysiology, treatment response, and temporal changes in brain activity according to natural course, additional studies with selected backgrounds and longitudinal studies are warranted. Third, fNIRS can only evaluate brain activity at the superficial level, and we only assessed the PFC region in this study. Since another study has reported that patients with FD have altered functional connectivity from the insula to the occipital lobe compared with healthy controls after fat ingestion [49], we evaluated the PFC only because of its better feasibility and greater involvement in the pathophysiology of DGBI than other cerebral areas. However, this non-invasive test using portable equipment can be performed in a natural posture and requires only minimal restraint without excluding individuals with non-removable metal objects in their bodies or tattoos. These advantages imply that this modality is highly useful for evaluating participants’ brain activity in a clinical setting.

In conclusion, patients with DGBI, particularly those with FD, disliked fatty food because of the strong association between fatty foods and abdominal symptoms. The brain activity in patients with DGBI differed from that in healthy controls. In particular, increased activity has been confirmed in the PFC in patients with FD. Establishing that patients with FD have different brain activity compared with that in healthy controls will benefit patients who have not received empathy for their experience from others because of the lack of significant abnormalities in other examinations. Therefore, data from fNIRS can be useful in investigating the pathophysiology of the disease and supporting objective diagnosis and assessment in daily clinical settings.

### Supplementary Information

Below is the link to the electronic supplementary material.

Supplementary file1 (PDF 545 KB)

Supplementary file2 (PDF 328 KB)

Supplementary file3 (PDF 57 KB)

Supplementary file4 (PDF 114 KB)

## Data Availability

The data for this study are available from the corresponding author if requested.
