# Supplementary figures and images for: Brain activity in response to food images in patients with irritable bowel syndrome and functional dyspepsia
Source: J Gastroenterol. 2023 Aug 12;58(12):1178–87. doi: 10.1007/s00535-023-02031-5 (PMC10657794; doi:10.1007/s00535-023-02031-5)

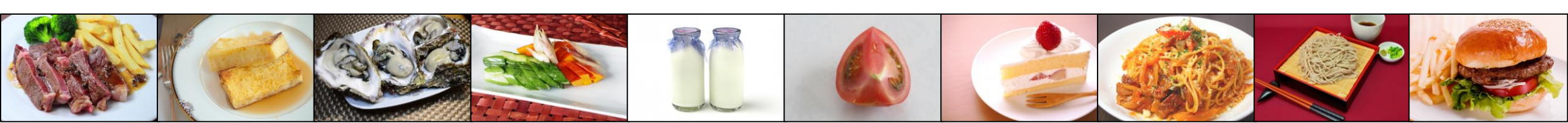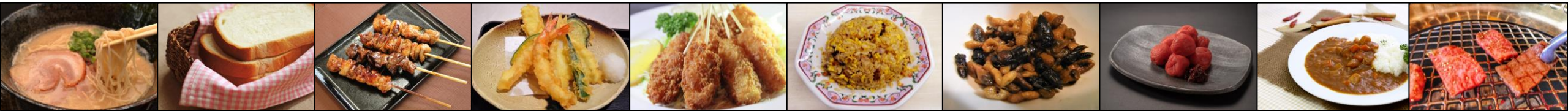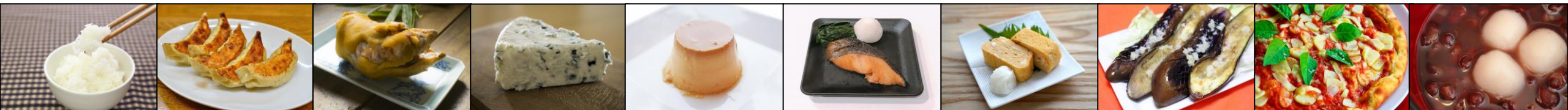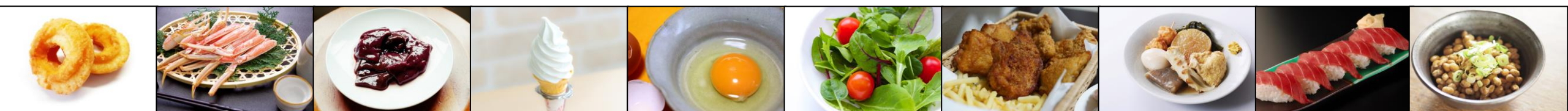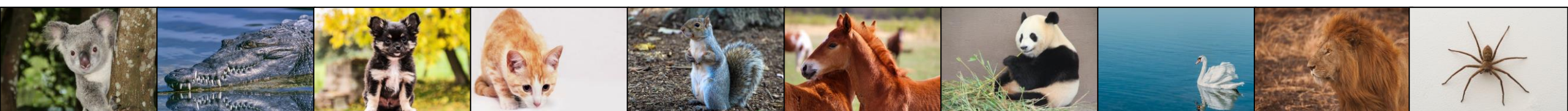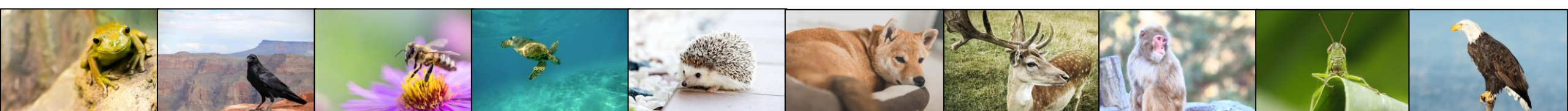

Supplement: Supplementary file 1 — Supplementary file1 (PDF 545 KB) [file 535_2023_2031_MOESM1_ESM.pdf]

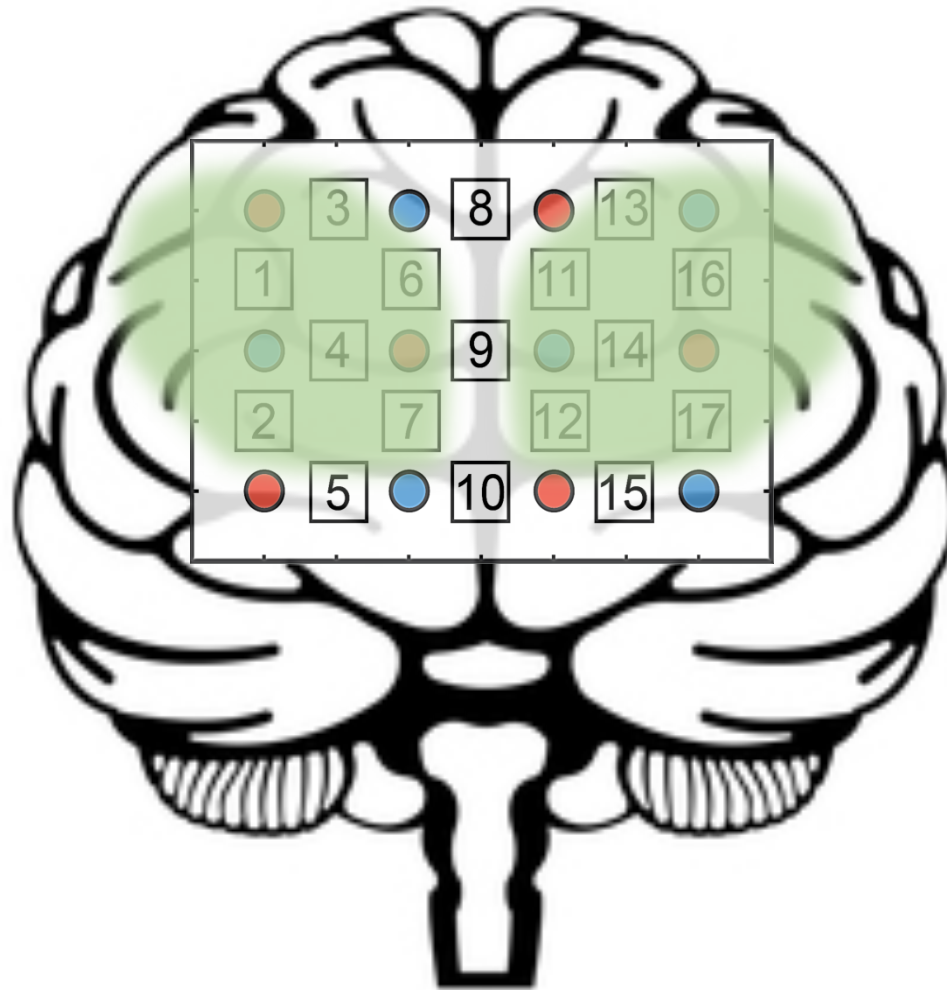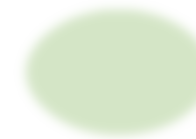

Estimated area of DLPFC

Supplement: Supplementary file 2 — Supplementary file2 (PDF 328 KB) [file 535_2023_2031_MOESM2_ESM.pdf]

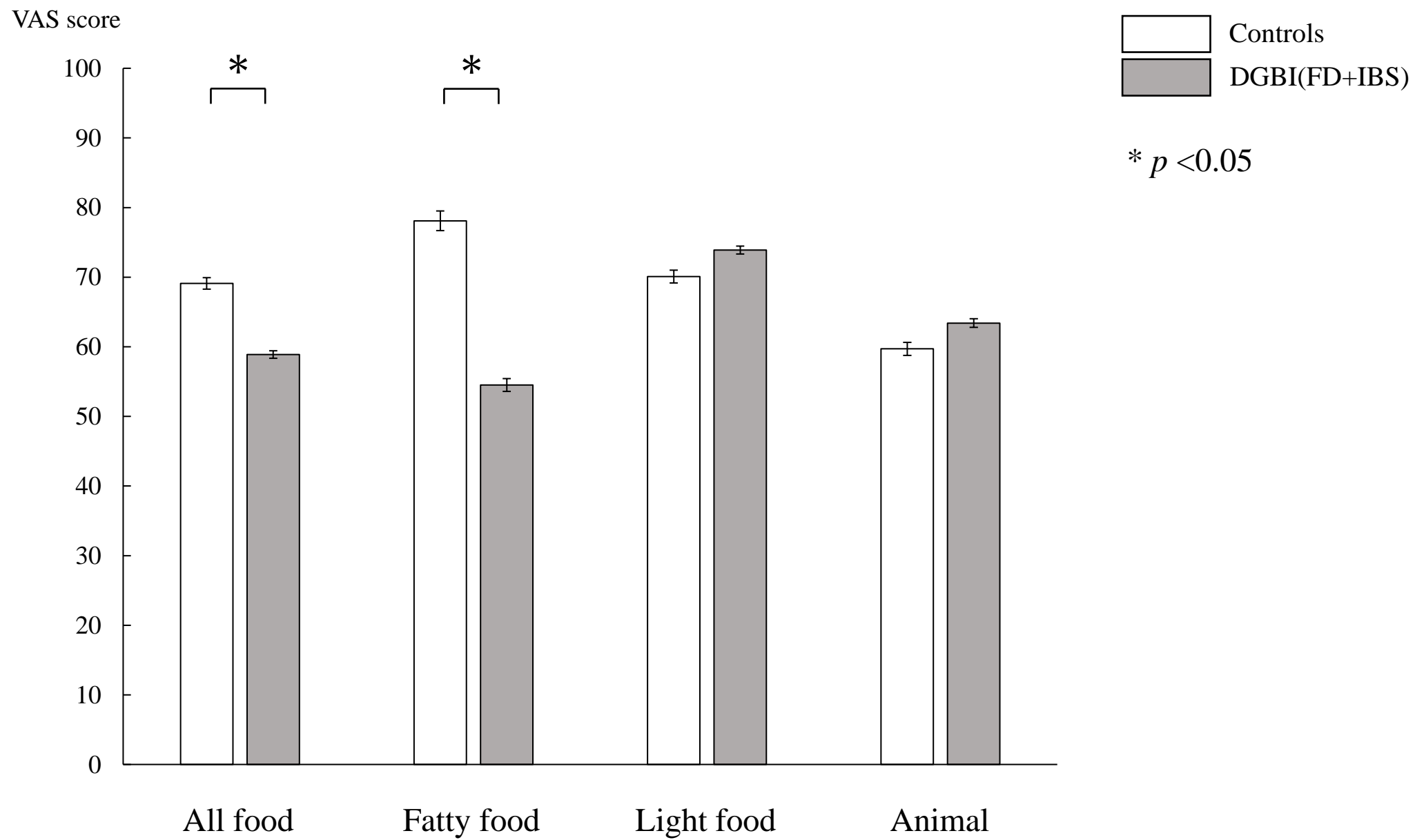

Supplement: Supplementary file 3 — Supplementary file3 (PDF 57 KB) [file 535_2023_2031_MOESM3_ESM.pdf]
