# Supplementary material for: Brain activity in response to food images in patients with irritable bowel syndrome and functional dyspepsia
Source: J Gastroenterol. 2023 Aug 12;58(12):1178–87. doi: 10.1007/s00535-023-02031-5 (PMC10657794; doi:10.1007/s00535-023-02031-5)
Supplement: Supplementary file 4 — Supplementary file4 (PDF 114 KB) [file 535_2023_2031_MOESM4_ESM.pdf]

Supplementary Table 1 Comparison of comorbidity and treatment among patients with functional dyspepsia, patients with irritable bowel syndrome, and healthy controls.

Variables	**Control (n=16)**	**FD (n=12)**	**IBS (n=13)**
Comorbidity (n [%])			
migraine	1 (6.2)	3 (25.0)	0 (0)
depression	0 (0)	0 (0)	1 (7.6)
Hypertension	1 (6.2)	0 (0)	1 (7.6)
Hyperlipidemia	1 (6.2)	1 (8.3)	1 (7.6)
Diabetes mellitus	1 (6.2)	0 (0)	1 (7.6)
FD			
EPS			2 (15.3)
PDS			1 (7.6)
Subtypes (n [%])			
EPS		4 (33.3)	
PDS		6 (50.0)	
Mixed		2(16.7)	
IBS-C			4 (30.7)
IBS-D			6 (46.2)
IBS-M			3 (23.1)
Treatment (n [%])			
Antacid		9 (75.0)	
Acotiamide		5 (41.7)	
Japanese herbal medicine		4 (33.3)	
Antiemetics		3 (25.0)	
Rebamipide		2(16.7)	
SSRI		2(16.7)	1 (7.6)
Laxative			3 (23.1)

Trimebutine maleate	3 (25.0)	2 (15.3)
Polycarbophil calcium		3 (23.1)
Antidiarrhea		4 (30.7)
Ramosetron hydrochloride		3 (23.1)
Mosapride		2 (15.3)

FD, functional dyspepsia; IBS, irritable bowel syndrome; n, number; EPS epigastric pain syndrome; PDS postprandial distress syndrome; IBS-C constipation predominant IBS; IBS-D diarrhea predominant IBS; IBS-M mixed IBS; SSRI, selective serotonin reuptake inhibitor
